# Cells under siege: Viral glycoprotein interactions at the cell surface

**DOI:** 10.1016/j.jsb.2011.03.016

**Published:** 2011-08

**Authors:** Thomas A. Bowden, E. Yvonne Jones, David I. Stuart

**Affiliations:** a Division of Structural Biology, University of Oxford, Wellcome Trust Centre for Human Genetics, Roosevelt Drive, Oxford OX3 7BN, United Kingdom; b Science Division, Diamond Light Source Ltd., Diamond House, Harwell Science and Innovation Campus, Didcot, Oxfordshire 0X11 0DE, United Kingdom

**Keywords:** Glycoprotein structure, Virus entry, Cell signaling, X-ray crystallography, Cell surface receptors, GAP, GTPase-activating protein, IPT, Ig-like plexins and transcription factors, HeV, Hendra virus, HeV-G, Hendra virus attachment glycoprotein, HNV, Henipavirus, HNV-G, Henipavirus attachment glycoprotein, NiV, Nipah virus, NiV-G, Nipah virus attachment glycoprotein, MACV, Machupo virus, PDB, protein databank, PSI, plexin-semaphrorin-integrin domain, r.m.s.d., root mean square deviation, Tf, transferrin, TfR1, transferrin receptor 1, SLAM, Signaling Lymphocytic Activation Molecule, SPINE, Structural Proteomics In Europe

## Abstract

As obligate parasites, viruses are required to enter and replicate within their host, a process which employs many of their proteins to hijack natural cellular processes. High resolution X-ray crystallographic analysis has proven to be an ideal method to visualize the mechanisms by which such virus-host interactions occur and has revealed the innovative capacity of viruses to adapt efficiently to their hosts. In this review, we draw upon recently elucidated paramyxovirus-, arenavirus-, and poxvirus-host protein complex crystal structures to reveal both the capacity of viruses to appropriate one component of a physiological protein–protein binding event (often modifying it to out-compete the host-protein), and the ability to utilize novel binding sites on host cell surface receptors. The structures discussed shed light on a number of biological processes ranging from viral entry to virulence and host antagonism. Drawn together they reveal the common strategies which viruses have evolved to interact with their natural host. The structures also support molecular level rationales for how viruses can be transmitted to unrelated organisms and thus pose severe health risks.

## 1 Introduction

Viruses have tremendous genetic diversity (Edwards and Rohwer, 2005), a property largely accounted for by rapid replication, frequent and unspecific mutations arising from error-prone polymerases, and an ability to recombine host genes into their own genome. The resulting capability of a single virion to generate a genetically diverse complement of progeny provides a simple mechanism by which virus-host cell interactions can rapidly become specialized for specific host ranges and tissues. Combined with the ability to ‘steal’ host proteins, this provides a powerful method by which viruses hijack natural host cell functions, facilitating processes such as viral attachment and antagonism of the host’s innate immune response (Bahar et al., 2011).

**Fig. 1:**
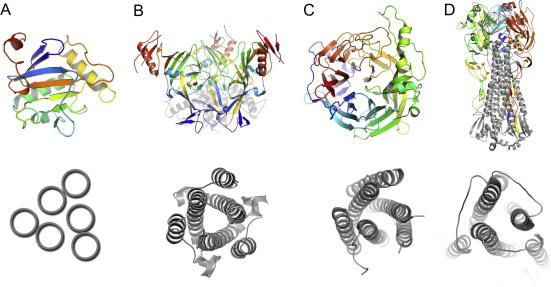
Contrasts in fold conservation between attachment and fusion glycoproteins from negative-sense, single-stranded RNA viruses. Receptor-binding attachment glycoproteins are shown above and the six-helix bundles from their cognate fusion glycoproteins below from (A) Machupo Arenavirus (PDB identification number 2WFO), (B) Ebola Filovirus (PDB ID 3CSY and 1EBO), (C) Nipah (above) and Parainfluenza type-III (below) Paramyxoviruses (PDB ID 2VSM and 1ZTM, respectively), and (D) Flu Orthomyxovirus (PDB ID 3LZG). Note for panel A, there are currently no known crystal structures of arenaviral fusion glycoproteins, in panel B, Ebola GP1 is shown with its non-covalently associated GP2 subunit (gray cartoon), and in panel D, Flu virus haemagglutinin is shown with the HA1 domain colored as a rainbow and the HA2 domain colored gray.

Crystallographic studies of viral proteins alone and in complex with their functional ligands have led to a greater appreciation of how the structurally dissimilar fold architectures resulting from viral genomic diversity can achieve analogous biological processes. Structural investigations of viral attachment glycoproteins, for example, have shown that enveloped viruses adopt a wide range of folds optimized for engagement of their cognate cellular receptors. These folds vary from the compact and novel α/β fold of *Arenaviridae* (Fig. 1A) (Abraham et al., 2010; Bowden et al., 2009a), to the trimeric GP1 ‘chalice’ of the *Filoviridae* (Fig. 1B) (Lee et al., 2008), the globular six-bladed β-propeller of the *Paramyxovirinae* (Fig. 1C) (Bowden et al., 2010a), the large trimeric hemagglutinin of the *Orthomyxoviridae* (Fig. 1D) (Weis et al., 1988; Wilson et al., 1981), and the highly glycosylated GP120 trimer of Lentiviruses in the *Retroviridae* (Wyatt et al., 1998; Zhu et al., 2003). It is noteworthy that the associated fusion glycoproteins from each of the above virus families, in contrast to the attachment glycoproteins, are similar in architecture and have all been grouped into the first of the three known structural classes of fusion proteins (Eschli et al., 2006; Lamb and Jardetzky, 2007; Lee et al., 2008) (Fig. 1AD). It has been suggested that these proteins are related (Kadlec et al., 2008).

Fusion and receptor-binding proteins are often synthesized on the same polypeptide and fold together to form complexes on the virus surface. As a result, these protein pairs have not evolved entirely independently. Nevertheless, receptor-binding attachment glycoproteins display much greater structural diversity than their fusion glycoprotein counter-parts. This is likely to stem from whether there is a functional requirement for a given viral protein to adapt to and interact with its hosts. Viral nucleoproteins and fusion glycoproteins (with the exception of the immunosuppressive segments of some fusion glycoproteins) (Avota et al., 2010; Cianciolo et al., 1985; Kleinerman et al., 1987; Volchkov et al., 2001; Yang et al., 2000), interact less-specifically with their host cell and have a relatively self-contained function (e.g. insertion into and merging of the viral and host envelopes or packaging of genomic material) which requires minimal adaption. Certain non-structural proteins and attachment glycoproteins, on the other hand, are examples of viral proteins which more often interact specifically with their host cell and adapt rapidly to cellular host factors.

Using recently elucidated paramyxovirus-, arenavirus-, and poxvirus-host complex crystal structures resulting from the Spine (Structural Proteomics In Europe) 2-Complexes initiative as examples (detailed in Table 1), we draw upon this second, adaptive group of viral proteins to reveal the varied strategies employed by viruses when interacting with their hosts. Setting these results in a broader context, through comparison with other structurally well established viral glycoprotein systems including HIV and Measles virus, we illustrate that viruses not only subvert binding sites that are used in natural physiological signaling processes, but can also exploit novel sites on host proteins previously not used as interaction surfaces.

## 2 Viral semaphorins and immune antagonism

Semaphorins comprise a family of cell surface signaling glycoproteins which, through binding to the family of plexin glycoprotein cell surface receptors, activate repulsive guidance pathways which are fundamental to a number of physiological processes including axon guidance, immune regulation and activation, and vascular development (Kruger et al., 2005; Suzuki et al., 2007). There are eight known classes of semaphorins: two found in invertebrates, five in vertebrates, and the eighth class in viruses which are known as ‘viral semaphorins’ (Comeau et al., 1998; Ensser and Fleckenstein, 1995). Whilst the ectodomains of cellular semaphorins contain C-terminal domain elaborations such as PSI (plexin, semaphorin and integrin) domains, immunoglobulin (Ig)-like domains, thrombospondin domains and PDZ-domain-binding sites which may or may not attach to the cell-surface, the N-terminal portion, comprising a plexin-binding sema-domain, is well conserved. The sema-domain is the only component found in viruses. Crystallographic studies, by ourselves and others, have shown that the human Sema3A and mouse Sema4D semadomains consist of a structurally conserved homodimer of seven-bladed β-propellers (1.7 Å root mean square deviation, r.m.s.d., for matching Cα atoms) (Antipenko et al., 2003; Janssen et al., 2010; Liu et al., 2010; Love et al., 2003; Nogi et al., 2010).

The domain architecture is conserved amongst the four classes (A–D) of vertebrate plexin type-I membrane glycoproteins and consists of an N-terminal, membrane distal sema-domain which is anchored to the membrane by PSI domains and IPT (Ig-like, plexins and transcription factors) domains (Bork et al., 1999). A GTPase-binding domain and a C-terminal segment GAP (GTPase-activating protein) domain constitute the intracellular portion of the glycoprotein and are responsible for activation of Rho family GTPase signaling pathways within the plexin-expressing cell (Kruger et al., 2005).

Immunoregulatory semaphorins including Sema3A, 4A, 4D, and 7A contribute to B cell mediated immunity (Sema4D), T cell activation and differentiation (Sema4A, Sema3A, and Sema4D), and inflammation (Sema7A) (Suzuki et al., 2008). Genomic sequencing of eukaryotic viruses has revealed that Poxviruses (e.g. Smallpox virus and Vaccinia virus) and alcelaphine herpes virus encode viral semaphorins which have been shown to modulate these processes (Comeau et al., 1998; Ensser and Fleckenstein, 1995; Suzuki et al., 2008). These viral encoded proteins were presumably ‘stolen’ from their host during the process of virus-host co-evolution (Suzuki et al., 2008). The glycoprotein A39R, encoded by Vaccinia virus, for example, shares highest sequence homology with Sema7A (∼30%) and undermines the host immune response by binding to plexinC1 (Comeau et al., 1998).

**Fig. 2:**
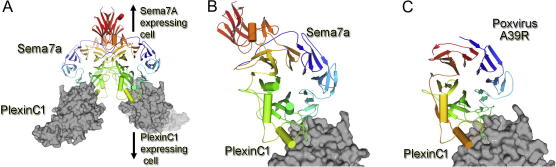
Poxviral appropriation of semaphorin-plexin interactions. (A) Crystal structure of Sema7a in complex with PlexinC1 (PDB ID 3NVQ). The PlexinC1 seven-bladed β-propeller domains are rendered as gray surfaces and the Sema7A dimer is shown as a cartoon with each protomer colored as a rainbow with the N-terminus in blue and the C-terminus in red. Close-up view of protein-protein interactions in the (B) Sema7a-PlexinC1 and (C) poxvirus A39R-PlexinC1 complex (PDB ID 3NVN) interfaces reveal nearly identical binding modes (colored as in panel A).

Recent crystallographic studies of semaphorin-plexin signaling complexes, in part within the activity of Spine2-Complexes, have enabled a detailed comparison of the mechanism by which natural- and viral-semaphorins bind to plexins. Structures of Sema7A-plexinC1 (Liu et al., 2010), Sema6A-plexinA2 (Janssen et al., 2010; Nogi et al., 2010), and Sema4D-plexinB1(Janssen et al., 2010), have revealed structurally similar signaling complexes, all composed of a Sema-plexin heterotetramer where each protomer of the semaphorin dimer binds to one plexin seven-bladed β-propeller sema domain (Fig. 2A and B). Elucidation of the crystal structure of vaccinia virus A39R in complex with plexinC1 demonstrates that poxviruses take advantage of an almost identical binding mechanism to that of physiological semaphorinplexin signaling, where both the viral and the cell semaphorin β-propellers bind to their cognate plexin β-propellers in a side-on orientation (Fig. 2C) (Liu et al., 2010). Furthermore, although the binding interface of the viral complex is less extensive to that observed in analogous physiological complexes (Sema7A-plexinC1), the binding strength of the A39R-plexinC1 interaction is significantly enhanced (*K*d of ∼10 nM versus ∼300 nM) (Liu et al., 2010). Whilst Sema-plexin binding affinities are delicately balanced to contribute to the complex interplay of interactions required for physiological functions, Vaccinia virus protein A39R can simply optimize a single interaction. These studies are an example of how genetic variability can give rise to mechanisms which enhance virus virulence and replication. In addition to incorporating genes from their host organism, these structures provide a molecular basis for how viruses can optimize their own proteins to override normal physiological interactions.

## 3 Henipavirus entry: The ephrin gateway

Nipah virus and Hendra virus compose the genus Henipavirus within the *Paramyxoviridae* family and are emergent and highly virulent bat-borne pathogens found in Africa, Australia, and South East Asia (Eaton et al., 2006; Wild, 2009). Oligomeric complexes of two glycoproteins extending outward from the viral envelope are required for efficient attachment (G glycoprotein) and fusion (F glycoprotein) into their respective host cells. During henipaviral attachment, the F glycoprotein is activated in a pH independent mechanism (Aguilar et al., 2009) to undergo classical class I fusion rearrangements which merge the host and viral membranes (Lou et al., 2006).

NiV-G and HeV-G (collectively referred to as HNV-G) are type-II transmembrane glycoproteins consisting of an N-terminal cytoplasmic tail, a short transmembrane region, an ectodomain stalk region, and a C-terminal receptor binding six-bladed β-propeller domain. HNV-G glycoproteins are important in determining the broad species and cellular tropism of these viruses as they have been observed to bind specifically with ephrinB2 and ephrinB3 cell surface receptors at nanomolar affinity (Bonaparte et al., 2005; Negrete et al., 2005, 2006). The sequences of ephrinB2 and ephrinB3 are well conserved amongst many vertebrate species including humans, bats, horses, and pigs (>95% sequence identity) (Bossart et al., 2008), and they are ubiquitously expressed in most human tissues due to their importance in fundamental bi-directional cell signaling processes such as osteogenesis, axon guidance, and vascular development (Hafner et al., 2004; Pasquale, 2005).

**Fig. 3:**
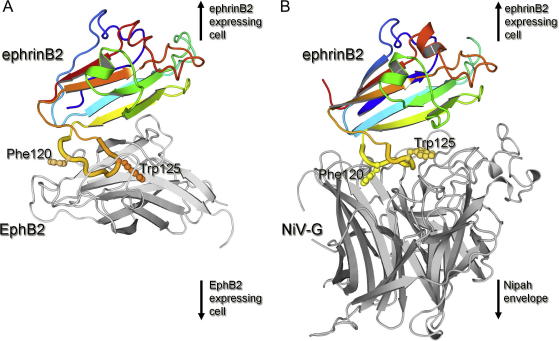
Differential utilization of the GH^ephrinB2^ loop by Eph receptors and Henipaviruses. (A) Crystal structure of EphB2 in complex with ephrinB2 (PDB ID 1KGY). (B) Crystal structure of NiV-G in complex with ephrinB2 (PDB ID 2VSM). For both panels, structures are shown as cartoons with EphB2 and NiV-G colored in gray and ephrinB2 colored as a rainbow with the N-terminus in blue and the C-terminus in red. The primary ephrinB2 interaction loop is highlighted with a thicker radius and the side-chains of residues important for both protein-protein interactions are labeled and shown in a ball and stick representation.

Crystal structures of ephrin ligands alone and in complex with their Eph receptors (determined by others and as part of Spine2-Complexes) have been invaluable for identifying the molecular specificity which underlies normal physiological signaling events. These studies reveal that the ephrin ectodomain forms a compact greek-key fold containing a ∼10 amino acid (GH) binding loop, which is predominantly responsible for Eph receptor binding through its insertion into the receptor binding cleft of the membrane-distal Eph receptor β-sandwich domain (Bowden et al., 2009b; Chrencik et al., 2006a, 2006b; Himanen et al., 2001, 2009, 2010, 2004; Nikolov et al., 2007, 2005; Qin et al., 2010; Seiradake et al., 2010; Toth et al., 2001) (Fig. 3A).

Site-directed mutagenesis of ephrinB2 and ephrinB3 confirmed that HNV-G subverts natural Eph receptor binding by also utilizing this GH loop during viral attachment (Negrete et al., 2006). However, rather than completely imitating the exact binding mode observed in physiological Eph-ephrin interactions, structural studies of HNV-G alone and in complex with ephrinB2 and ephrinB3 show that the GH loop is plastic and undergoes a unique rearrangement that allows it to bind to the top center portion of the HNV-G β-propeller (Fig. 3B) (Bowden et al., 2008a; Xu et al., 2008). As the GH loop is well conserved between many vertebrate species including bats and humans, this observation provides a molecular level rationale for Henipaviral zoonosis (Bowden et al., 2008a). The conformational changes observed in the ephrins, in addition to those occurring to the HNV-G β-propeller upon binding (Bowden et al., 2010b, 2008a, 2008b; Xu et al., 2008), result in a protein-protein interface which is similarly tight (nanomolar affinity) but more extensive than physiological Eph-ephrin interactions (an HNVG-ephrinB2 interface of approximately 2700 Å^2^ compared to an average of 2200 Å^2^ buried surface area for an Eph-ephrin complex). Despite differences in the extent of these interactions, the protein-protein interfaces in both sets of structures are dominated by hydrophobic contacts between aromatic sidechains of ephrinB2 and ephrinB3 (e.g. Phe120^ephrinB2^ and Trp 125^ephrinB2^) with binding pockets on the physiological Eph and viral HNV-G glycoproteins. Such binding surfaces are reminiscent to the hydrophobic contacts observed in structures of CD4 in complex with MHC class II and HIV GP120 glycoproteins. The interfaces in both CD4 complexes are dominated by the insertion of Phe43^CD4^ into hydrophobic cavities present on the MHC and HIV GP120 glycoproteins (Kwong et al., 1998; Wang et al., 2001).

The structural properties observed in henipavirus-ephrin interactions, in addition to those observed in the attachment glycoproteins from other paramyxoviruses (e.g. Measles hemagglutinin in complex with cell surface SLAM (Hashiguchi et al., 2011) and CD46 (Santiago et al., 2010)), underscore the adaptability of the viral six-bladed β-propeller scaffold (Bowden et al., 2010a; Stehle and Casasnovas, 2009), and in a broader context, the innovative ability of viruses to alter their glycoprotein repertoire to target new receptors and hosts.

## 4 Convergent viral attachment through transferrin receptor targeting

**Fig. 4:**
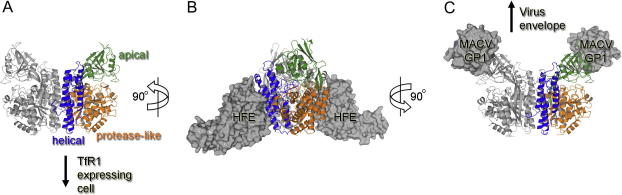
Contrasting modes of TfR1-host and-virus interactions. (A) Crystal structure of the unbound TfR1ectodomain (PDB ID 1CX8). One TfR1 protomer of the dimer is colored with the helical domain in blue, the protease-like domain orange, and the apical domain green. (B) Crystal structure of hereditary haemochromatosis protein HFE in complex with human TfR1 (PDB ID 1DE4). HFE molecules are rendered as gray surfaces and bind to helical TfR1 domains with a 2:2 stoichiometry. TfR1 is rotated by 90° along the vertical axis with respect to panel A. (C) Crystal structure of Machupo virus attachment glycoprotein GP1 (MACV GP1) in complex with human TfR1 (PDB ID 3KAS). MACV GP1 molecules are rendered as gray surfaces and bind to apical TfR1 domains in a 2:2 stoichiometry. TfR1 is shown in the same orientation as in panel A.

The transferrin receptor (TfR1) is a type-2 membrane glycoprotein which regulates the cellular uptake of iron through binding to its ligand, transferrin (Tf) and is almost ubiquitously expressed in different human tissues (Ponka and Lok, 1999). Upon binding to mono-ferric or di-ferric Tf, the TfR1-Tf complex is internalized through clathrin-dependent endocytosis and later is freed from TfR1 in acidic compartments (Ponka and Lok, 1999). TfR1 exists as a disulfide-linked dimer which consists of an N-terminal cytoplasmic domain, a transmembrane region and a 650 amino acid ectodomain. A major portion of the TfR1 ectodomain has been crystallized and shown to consist of a protease-like domain, a helical domain and an apical domain (Fig. 4A) (Bennett et al., 2000; Lawrence et al., 1999). Structures of TfR1 in complex with Tf and HFE, a membrane glycoprotein associated with hereditary haemochromatosis (Bomford, 2002; Lebron et al., 1999), have been elucidated by cryo-electron microscopy (Cheng et al., 2004) and crystallography (Bennett et al., 2000), respectively. HFE can compete with Tf for binding and both complex structures revealed a 2:2 stoichiometry (Fig. 4B). In these structures, the Tf and HFE binding sites overlap at the membrane proximal TfR1 helical domain (Fig. 4B) and are extensive; the crystal structure of the TfR1–HFE complex revealed the occlusion of approximately 2000 Å^2^ of solvent accessible surface.

In addition to its importance in iron delivery into cells, TfR1 has emerged as an entry receptor for a number of important pathogens including mouse mammary tumor virus (Ross et al., 2002), canine and panleukopenia feline parvoviruses (Parker et al., 2001), and New World hemorrhagic fever arenaviruses (Radoshitzky et al., 2007). These viruses differ markedly in properties: canine and panleukopenia feline parvoviruses are small (26 nm in diameter), icosohedral, single-stranded DNA viruses that do not contain a lipid bilayer envelope, whilst mouse mammary tumor virus and New World hemorrhagic fever arenaviruses are large, plieomorphic, enveloped viruses (∼100 and 120 nm in diameter, respectively) which contain single-stranded RNA genomes. Structural and functional studies of these viruses have shown that they attach to sites on TfR1 which do not overlap with the physiological Tf and HFE binding sites (Radoshitzky et al., 2007). The structure of the Machupo virus attachment glycoprotein, GP1, determined as a part of the Spine2-Complexes project, revealed a novel protein fold (Bowden et al., 2009a). A subsequent crystal structure of GP1 in complex with TfR1 has revealed an extensive binding site occluding over 1900 Å^2^ of solvent accessible surface at the tip of the membrane distal TfR1 apical domain (Fig. 4C) (Abraham et al., 2010). Similarly, functional and electron microscopic data suggest mouse mammary tumor virus and canine and feline parvoviruses also utilize the TfR1 apical domain for attachment (Goodman et al., 2010; Hafenstein et al., 2007; Palermo et al., 2003; Wang et al., 2006).

It has been suggested that this TfR1 ‘viral binding patch’ is used as it is remote from the known physiological Tf and HFE cellular binding sites and unlikely to disturb TfR1 endocytosis (Goodman et al., 2010). Given this hypothesis and the innate ability of viruses to evolve rapidly, it is not surprising that these diverse viruses have independently evolved similar molecular mechanisms to rely on the TfR1 cell surface receptor for virus entry.

## 5 Concluding remarks

The rapidity of sequence changes in viral genomes is fundamental for the survival of many viruses. It enables co-evolution with natural host reservoirs as well as opportunities to adapt to and infect new hosts. Such genetic variability is thus extremely problematic from a biomedical perspective. For example, poxviruses such as smallpox virus have used these properties to ‘steal’ and optimize host cell genes such as the plexinC1 interacting semaphorin, A39R, for antagonism of the host immune system. Emergent RNA viruses such as henipa- and arena-viruses, on the other hand, have relied on genetic diversity to develop very different attachment glycoprotein folds which can be used to bind to a variety of host cell surface receptors. The methodological advances in eukaryotic cell expression for macromolecular crystallography developed in Spine (Aricescu et al., 2006; Chang et al., 2007) have been invaluable for understanding the molecular basis of these virus-host cell interactions.

In this review, recently elucidated virus-host crystal structures, many of which have emerged from the Spine2-Complexes project, have shown how viruses both appropriate existing cellular interactions (e.g. A39R binding to PlexinC1 and henipavirus attachment to ephrins) as well utilize novel modes for host-interaction (e.g. arenaviral attachment to TfR1). These structural insights when drawn together reveal common molecular-level strategies which viruses have evolved to interact with their natural host and result in a danger to human and animal health.

## Figures and Tables

**Table 1:** Relevant structures solved under the European Spine initiatives.

Structure	Reference	PDB accession code
Semaphorin4D_Ecto_^a^	Love et al. (2003)	1OLZ
PlexinA2_D1-4_^b^	Janssen et al. (2010)	3OKT
Semaphorin6A_Ecto_	Janssen et al. (2010)	3OKW
Semaphorin4D_Ecto_-PlexinB1_D1-2_	Janssen et al. (2010)	3OL2
Semaphorin6A_Ecto_-PlexinA2_D1-4_	Janssen et al. (2010)	3OKY
EphA4_LBD_^c^	Bowden et al. (2009b)	2WO1
EphA4_LBD_-ephrinB2_RBD_^d^	Bowden et al. (2009b)	2WO2
EphA4_LBD_-ephrinA2_RBD_	Bowden et al. (2009b)	2WO3
EphA2_Ecto_	Seiradake et al. (2010)	2X10
EphA2_Ecto_-ephrinA5_RBD_	Seiradake et al. (2010)	2X11
NiV-G	Bowden et al. (2008b)	2VWD
HeV-G	Bowden et al. (2010b)	2X9M
NiV-G-ephrinB2	Bowden et al. (2008a)	2VSM
HeV-G-ephrinB2	Bowden et al. (2008a)	2VSK
MACV-GP1	Bowden et al. (2009a)	2WFO

a Ecto, entire ectodomain.

b D1–4, domains 1–4.

c LBD, ligand binding domain.

d RBD, receptor binding domain.
